# Graph Theory Identifies Autistic Patterns in the Prefrontal Circuit of a Mouse Model of Autism

**DOI:** 10.21203/rs.3.rs-8725198/v1

**Published:** 2026-02-03

**Authors:** Rongsong Liu, Yan Zhang, Mallory Lai, Viyaleta Davydzenka, Casey Moffitt, Nathaniel England, Giovanni Barbera, Rong Chen, Da-Ting Lin, Yun Li

**Affiliations:** 1. Department of Mathematics and Statistics, University of Wyoming, 1000 E. University Ave, Laramie, WY 82071, USA; 2. Department of Zoology and Physiology, University of Wyoming, 1000 E. University Ave, Laramie, WY 82071, USA; 3. Intramural Research Program, National Institute on Drug Abuse, National Institutes of Health, 333 Cassell Drive, Baltimore, MD 21224, USA; 4. Department of Diagnostic Radiology and Nuclear Medicine, University of Maryland School of Medicine, 100 N. Greene St., Baltimore, MD 21201, USA; 5. The Solomon H. Snyder Department of Neuroscience, Johns Hopkins University School of Medicine, 725 N. Wolfe Street, Baltimore, MD 21205; 6. Present address: National Institute on Drug Dependence and Beijing Key Laboratory of Drug Dependence, Peking University, Beijing, China; 7. These authors contribute equally to the work.

## Abstract

As a well-developed branch of mathematics, graph theory provides unique tools to quantifiably assess various properties of complex networks. Applied to brain circuits, network-level analyses can illustrate disruptions to brain organization that yield both mechanistic and diagnostic insights. Previously, graph theory has been used with functional magnetic resonance imaging datasets to quantify connections among different brain regions, readily capturing the macroscopic-scaled differences in brain networks between healthy and Alzheimer’s subjects. Here, we applied graph theory on the microscopic scale, using miniscope-based calcium imaging from the freely behaving wild type (WT) and *Shank3*^*fx*^ mice (a mouse model of autism), and compared functional connections among individual neurons in the prefrontal microcircuits during social behavior. We demonstrated that *Shank3*^*fx*^ mice displayed reduced neural activity, less-integrated network, and fewer network changes in the prefrontal microcircuits between the presence and absence of social targets. Furthermore, we employed machine learning to test whether graph-theoretic metrics extracted from the prefrontal microcircuits could be predictive of genotype and genotype-associated social behavior difference between *Shank3*^*fx*^ and WT mice. Our results indicate a strong link between altered prefrontal microcircuits and social behavior deficits in an autism mouse model, highlighting prefrontal microcircuitry as a potential diagnostic and therapeutic target for autism.

## Introduction

Autism Spectrum Disorder (ASD) is a complex neurodevelopmental condition that occurs in all racial and ethnic groups^1, 2^. The average prevalence of ASD is 1.7%, occurring four times more commonly among boys than girls^1, 2^. People with ASD exhibit differences in social behavior, difficulty in social communication, and repetitive behaviors^3^. Although the majority of ASD cases are idiopathic, 20–30% cases are linked to genetic variants. These include three classes: single gene mutations, copy number variants, and accumulation of polygenic risk factors^4, 5, 6^. Little is known about the underlying neurological basis of ASD and no cure is currently available.

Mouse models based on single ASD-associated genes, as well as copy number variants, have been developed^7, 8, 9, 10^. Among those single genes that have been implicated in ASD, several are responsible for synaptic development, including neurexin, neuroligin, shanks, and cadherins^5, 10^. Shank3, a synaptic scaffolding protein enriched at the postsynaptic density of excitatory synapses, is one of the most well-characterized ASD-associated genes^11^. Consistent with this, Shank3 knockout mice and monkeys exhibit reduced social interaction and prolonged self-grooming in mice^12, 13, 14^.

Differences in the prefrontal cortex function has been implicated in both human patients and rodent models for ASD^10, 15, 16, 17^, with functional imaging studies suggesting that the prefrontal cortex is heavily involved in social cognition^18, 19^. In rodents, the medial prefrontal cortex connects reciprocally with most cortical and subcortical brain regions, and carries critical functions including attentional processes, top-down emotion regulation, decision-making, working memory, and executive function. Lesions in the medial prefrontal cortex of rodents cause differences in social behavior^20^, and optogenetic manipulation of the excitatory/inhibitory balance in this brain region altered social exploration^21^.

Understanding the coordinated activity of the prefrontal cortex is essential for linking neural structure to function and behavior, as neuronal circuits support behavior through coordinated interactions that define network organization. Graph theory is a well-developed field and provides mathematical tools for assessing the properties of complex networks^22, 23, 24^. It has been successfully applied to macroscopic-scale brain network datasets obtained with functional MRI for identification of brain network changes among different brain regions in Alzheimer’s cases^25, 26^. Here, we focused on leveraging graph theory on a microscopic scale neural network to understand differences in functional connectivity in the prefrontal cortex between wild-type (WT) and *Shank3*^*fx*^ mice, a previously established ASD mouse model^12^.

To study functional connectivity among neurons on a microscopic scale, advanced techniques that enable neuron identification and continuous recordings of individual neural activity changes in a certain brain region from freely behaving animals, are required. Miniscope based *in vivo* calcium imaging, identifies active neurons through calcium-dependent fluorescence changes, enabling hundreds of individual neurons to be monitored as spatially distinct sources of activity changes during animal’s free behavior^27, 28, 29^. Once identified, active neurons can be modeled as nodes in a graph, with significant functional interactions, determined by thresholding pairwise correlations in calcium activity, as edges. These binarized connectivity graphs can enable comparisons of functional connectivity across various conditions using graph-theoretic metrics.

We performed miniscope *in vivo* calcium imaging in the prelimbic cortex, a subregion of the medial prefrontal cortex, during social behavior tasks for wild type mice and homozygous *Shank3*^*fx*^ mice. We demonstrated that *Shank3*^*fx*^ mice displayed abnormalities in sociability and preference for social novelty, along with reduced neural activity in the prelimbic cortex. To unravel the underlying circuit mechanisms correlated with social behavior differences in ASD, we applied graph theory analytical tools to compare the functional connectivity within the prelimbic microcircuits between *Shank3*^*fx*^ and wild type mice during their social behavior tasks. We demonstrated that *Shank3*^*fx*^ mice displayed a less integrated network and fewer network changes in the prefrontal microcircuits between the presence and absence of social targets, as evidenced by a lower clustering coefficient (*C*_*l*_), a longer average shortest path length (*L*_*g*_), and smaller spectral distances (*s*) between social conditions. Furthermore, machine learning was used to test whether graph theory metrics extracted from prelimbic microcircuit could readily predict genotype and genotype-associated social behavior differences between *Shank3*^*fx*^ and WT. Finally, feature selection and ablation analyses verified neural activity and spectral distances (*s*) as graph-theoretic indices carrying strong predictive signal. Our results highlighted the links between dysfunctional prefrontal microcircuits and social behavior deficits in autism. Insights from this study will pave the way for developing novel diagnostic and therapeutic strategies for autism.

## Results

### *Shank3*^*fx*^ mice displayed abnormalities in social behavior and neural activity in the prelimbic cortex

**Figure 1.**
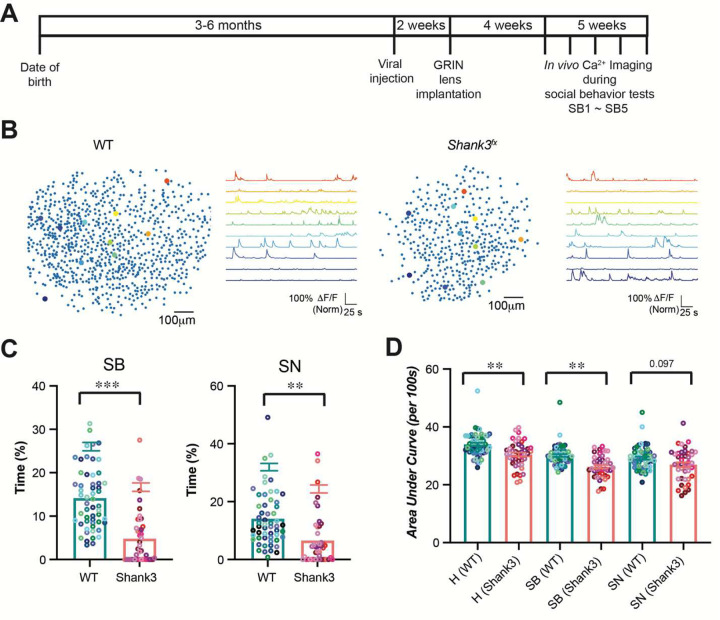
*Shank3*^*fx*^ mice displayed abnormal social behaviors and lower calcium activity. **(A)** Timeline of experiment. **(B)** Representative spatial maps from WT and *Shank3*^*fx*^ mice. Each dot represents one CaImAn-identified neuron. Colored dots indicate neurons with representative calcium traces. Color-matched calcium traces (normalized *ΔF/F*) from those highlighted corresponding neurons. **(C)** Bar graphs showing quantitative comparisons of percentage time on exploring social target during the SB stage (left panel) and on exploring novel social target during the SN stage (right panel). 9 WT and 11 *Shank3*^*fx*^ mice, SB1-SB5, Linear Mixed-Effects Model test. **(D)** Bar graphs showing quantitative comparisons of calcium activity (*AUC*/100*s*) between WT and *Shank3*^*fx*^ mice. Bar graphs represent mean ± standard error of the mean. 9 WT and 11 *Shank3*^*fx*^ mice, SB1-SB5, Linear Mixed-Effects Model test. *, p < 0.05; ****, p < 0.0001.

To unravel prefrontal microcircuit changes associated to autism, we performed miniscope *in vivo* calcium imaging in the prelimbic cortex (a subregion of the medial prefrontal cortex) during a three-stage social behavior test, from eleven wild type (WT) mice and nine homozygous *Shank3*^*fx*^ mice, a previously established ASD mouse model^12^. The social behavior test consists of habituation (H), sociability (SB), and social novelty (SN) stages, each lasting 10 minutes. Following the exploration of two empty containers during the H stage, mice were tested for sociability in the SB stage by comparing exploration time between a social target and an empty container. Subsequently, preference for social novelty was assessed during the SN stage by comparing exploration time between a novel social target and a relatively familiar one. We repeated *in vivo* calcium imaging on the same mice for five successive social behavior tests (Figure 1A), and obtained cell maps from the prelimbic cortex of these WT and *Shank3*^*fx*^ mice (Figure 1B and **Supplementary Figure S1)**. The cell maps captured the spatial distribution of individual active neurons within the imaging field and provided the foundation to functionally constructing session-specific neuronal networks.

During the five social behavior tests, WT mice consistently spent more time exploring the social target than the empty container in the SB stage, and spent more time exploring the novel social target than the relatively familiar one in the SN stage, demonstrating normal sociability and a preference for social novelty **(Supplementary Figure S2A)**. However, homozygous *Shank3*^*fx*^ mice displayed no preference in exploring social targets during both SB and SN stages, indicating impaired sociability and preference for social novelty **(Supplementary Figure S2B)**. Relative to WT mice, homozygous *Shank3*^*fx*^ mice displayed substantially less time exploring the social target during the SB stage and the novel social target during the SN stage (Figure 1C). Along with the social behavior abnormalities, *Shank3*^*fx*^ mice exhibited decreases in neural calcium activity of the prelimbic cortex, measured by the area under curve (*AUC*) of calcium traces for H, SB, and SN stages, relative to WT mice (Figure 1D).

### Graph theory indices quantifiably distinguished differences between *Shank3*^*fx*^ and WT prelimbic microcircuits

**Figure 2.**
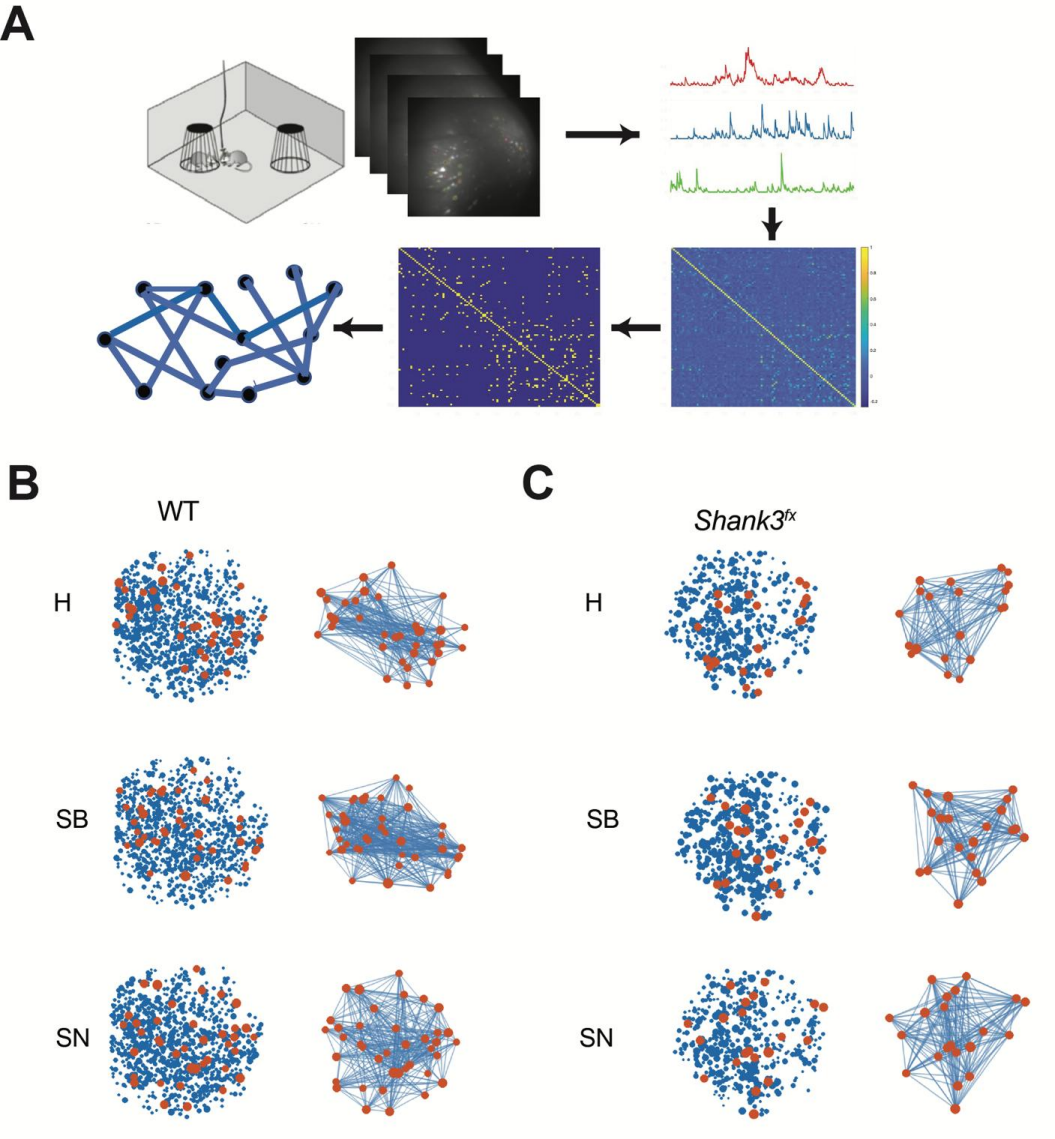
Prelimbic microcircuits are small-world networks. **(A)** Schematic diagram summarizes the pipeline from miniscope calcium imaging to calcium traces of individual active neurons, binarized connectivity, correlation matrices, and corresponding graphs with nodes and edges. The resulting networks exhibited characteristic features of small-world organization, including clustered local connectivity and the presence of highly connected nodes (i.e., hub neurons). **(B)** Representative examples from WT mice showing neuronal cell maps (left) and corresponding hub-neuron subnetworks (right) during H, SB, and SN stages. Each point represents a neuron, with hub neurons highlighted in red and non-hub neurons shown in blue; hub-neuron subnetworks display only hub nodes and their functional connections. **(C)** Representative examples from *Shank3*^*fx*^ mice shown in the same format as in (**B**). Compared with WT microcircuits, *Shank3*^*fx*^ networks exhibit altered hub-neuron distributions and reduced qualitative reorganization of hub connectivity across behavioral stages.

To construct a functional microcircuit graph based on *in vivo* calcium imaging in the prelimbic cortex, all identified active neurons were recognized as nodes. Significant functional interactions were treated as edges, determined by thresholding pairwise correlations among active neurons (nodes). These binarized connectivity graphs would enable comparisons of functional connectivity across various conditions using graph-theoretic metrics (Figure 2A). In these graphs, highly connected nodes were identified as hub neurons (see Methods). To visualize the spatial organization of hub neurons across behavioral stages, we illustrated hub-neuron cell maps and corresponding hub-neuron subnetworks from representative WT and *Shank3*^*fx*^ mice during H, SB, and SN stages (Figure 2B–C and **Supplementary Figure S3**).

We demonstrated that the prelimbic microcircuits from both WT and *Shank3*^*fx*^ mice exhibited network organization consistent with features commonly associated with small-world structure^30, 31^. In both WT and *Shank3*^*fx*^ microcircuits, hub neurons were distributed across the imaging field and exhibited visually apparent stage-dependent rewiring, characterized by gain and loss of hub-centered connections and changes in local neighborhood structure across behavioral stages. These stage-dependent differences were apparent both in the spatial arrangement of hub neurons and in the structure of their associated connectivity patterns.

To quantifiably comparing the functional connectivity within the prelimbic microcircuits between *Shank3*^*fx*^ and wild type mice during their social behavior tests, we next applied graph theory analytical tools to quantify key aspects of network organization (**Supplementary Table S1**). Mathematically, graph-theoretic indices include the average shortest path length (*L*_*g*_), the mean local clustering coefficient (*C*_*l*_), and the global clustering coefficient (*C*_*g*_). These metrics quantify clustering and global integration, reflecting how efficiently information propagates across the network. While such metrics characterize static topological properties, they are often insufficient to quantify holistic changes in network architecture across behavioral states or experimental conditions. To address this limitation, we also calculated spectral distance (*s*), to provide a complementary framework that captures global differences in network organization across behavioral stages. Biologically, smaller spectral distances indicate similar connectivity architectures that support comparable modes of signal propagation across stages, whereas larger distances reflect substantial reconfiguration of network topology and altered pathways of information flow across stages. Thus, spectral distance provides a biologically meaningful index for quantifying stage-dependent reorganization of neuronal microcircuits and enables direct comparison of network-level dynamics across conditions.

**Figure 3.**
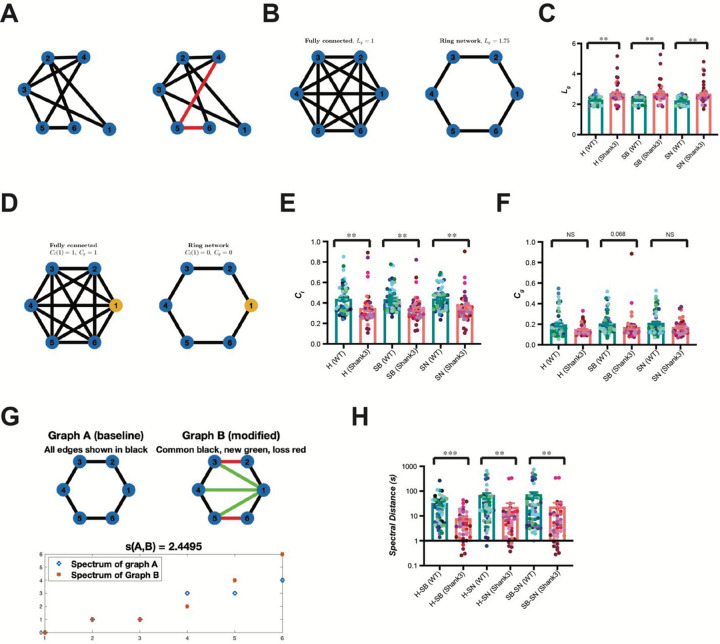
*Shank3*^*fx*^ mice displayed abnormal prefrontal network connectivity. **(A)** Schematic illustration for shortest-path metric *L*_*g*_. Left: an example graph with six nodes. Right: shortest path from the node 1 to node 6 (red) is indicated. **(B)** Characteristic path length (*L*_*g*_) in two networks of six neurons. In the highly interconnected network (left), every neuron is directly linked to all others, resulting in the shortest possible average path length (*L*_*g*_ = 1.0). In the medium connected network (right), neurons are arranged in a ring, requiring information to traverse intermediate nodes, which increases the average path length (*L*_*g*_ = 1.8). *L*_*g*_ quantifies the efficiency of information transfer across the network. **(C)** Bar graphs showing quantitative comparisons of *L*_*g*_ between WT and *Shank3*^*fx*^ mice. **(D)** Illustration of the local clustering coefficient (*C*_*l*_) and global clustering coefficient (*C*_*g*_) in two networks of six neurons. The highlighted orange neuron (node 1) is used to illustrate local clustering. In the highly interconnected network (left), the neighbors of node 1 are all connected to each other, giving *C*_*l*_(1) = 1.0, and the overall network has a high global clustering coefficient (*C*_*g*_ = 1.0). In the medium connected network (right), the neighbors of node 1 are not connected to each other, giving *C*_*l*_(1) = 0and resulting in a low global clustering coefficient (*C*_*g*_ = 0). This demonstrates how *C*_*l*_) captures the density of connections within a node’s neighborhood, while *C*_*g*_) summarizes this property across the entire network. **(E)** Bar graphs showing quantitative comparisons of *C*_*l*_ between WT and *Shank3*^*fx*^ mice. **(F)** Bar graphs showing quantitative comparisons of *C*_*g*_ between WT and *Shank3*^*fx*^ mice. **(G)** Spectral distance between two graphs with the same node set. Top left (Graph A): baseline ring on nodes 1–6. Top right (Graph B): modified network; new edges shown in green, lost edges in red (dashed), and unchanged edges in light gray. Bottom: Sorted eigenvalues of the Laplacian matrices for Graphs A and B. The spectral distance was computed as the Euclidean norm of the difference between their Laplacian eigenvalue vectors, excluding the zero mode. A larger spectral distance indicates a greater divergence in global network structure and, biologically, a stronger reorganization of neural microcircuit connectivity underlying differences in information integration and communication flow. **(H)** Bar graphs showing quantitative comparisons of spectral distance *s*(*H* − *SB*), *s*(*H* − *SN*), *s*(*SB* − *SN*) between WT and *Shank3*^*fx*^ mice. Bar graphs represent mean ± SEM. 9 WT and 11 *Shank3*^*fx*^ mice, SB1-SB5, Linear Mixed-Effects Model test. *, *p* < *0.05*; ****, *p* < *0.0001*; NS, not significant.

Relative to WT, *Shank3*^*fx*^ prelimbic microcircuits exhibited a marked increase in average shortest path length (*L*_*g*_) (Figure 3A–C) and a smaller local clustering coefficient (*C*_*l*_) (Figure 3D, E) at each behavioral stage (H, SB, and SN), indicating reduced network integration and local cohesiveness. In contrast, the global clustering metric (*C*_*g*_) did not differ significantly between genotypes across behavioral states, with only a trend toward reduction in the SB stage (Figure 3F). Importantly, in addition to these network feature changes identified within each behavioral stage, the spectral distance (*s*) between any two behavioral stages, were considerably larger in WT than those in *Shank3*^*fx*^ microcircuits (Figure 3G–H), indicating that WT prelimbic connectivity matrices reconfigured more drastically across behavioral stages involving the presence and absence of social targets.

### Calcium activity and graph theory indices predicted genotype and behavior outcomes

**Figure 4:**
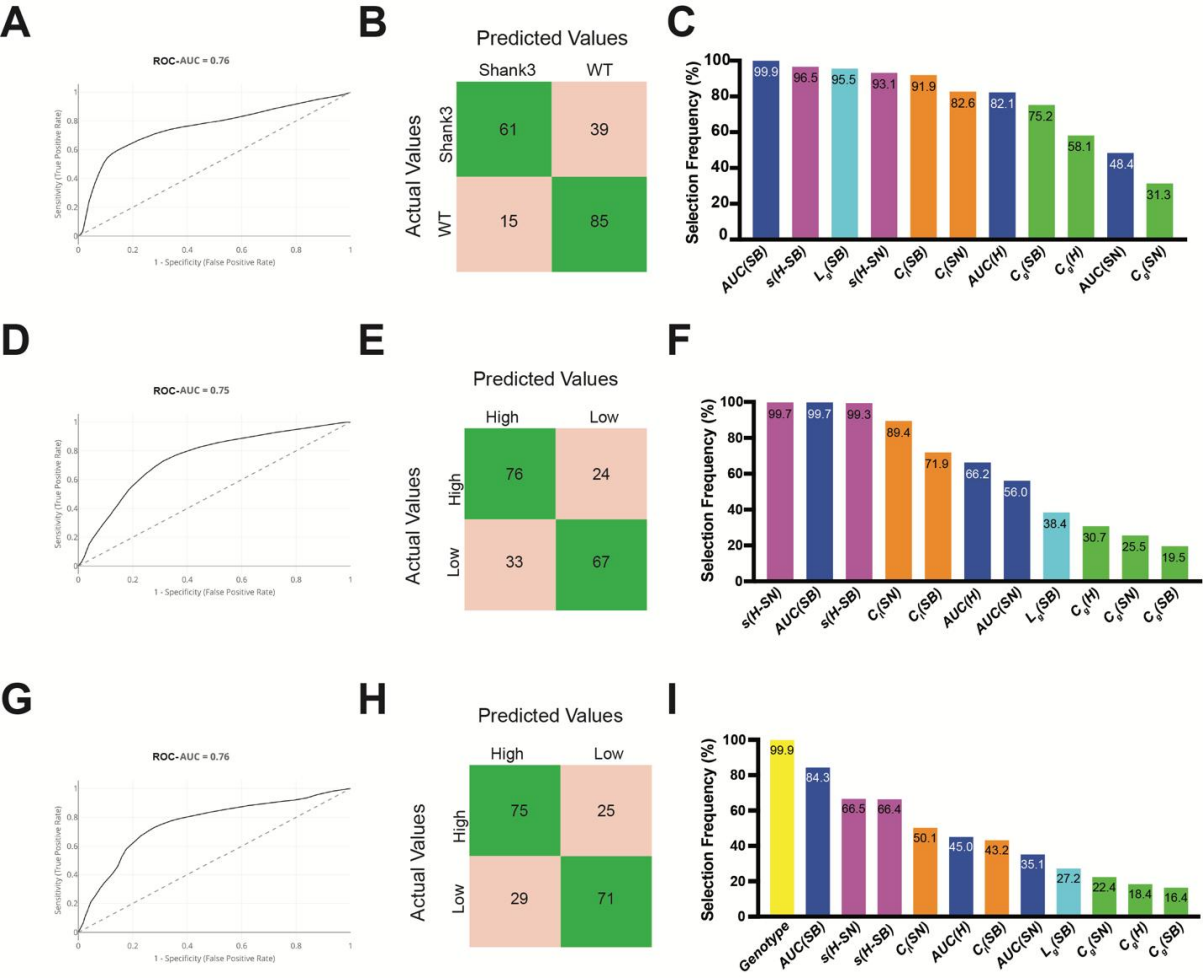
Genotype and social behavior prediction from graph-theoretic network indices. **(A)** ROC curve (ROC-AUC = 0.76) and confusion matrix averaged across all *1,980* outer-loop test sets from **Model 1** (TP = *61*, FN = *39*, FP = *15*, TN = *85*). **(B)** Performance summary of Model 1 at this threshold: sensitivity = *0.61*, specificity = *0.85*, PPV = *0.80*, NPV = 0.69. **(C)** Feature selection frequency across outer splits; bars show the percentage of splits in which each metric was retained by the final model 1. **(D)** ROC curve (ROC-AUC = 0.75) and confusion matrix averaged across all *1,980* outer-loop test sets from **Model 2** (*TP = 76, FN = 24, FP = 33, TN = 67*). **(E)** Performance summary of Model 2 at this threshold: sensitivity = *0.76*, specificity = *0.67*, PPV = *0.70*, NPV = *0.74*. **(F)** Feature selection frequency across outer splits for the genotype (blue) and behavior (hatched red) models. Bars indicate the percentage of splits in which each metric was retained by the final model. **(G)** ROC curve (ROC-AUC = 0.76) and confusion matrix averaged across all 1,980 outer-loop test sets from **Model 3** (TP = *75*, FN = *25*, FP = *29*, TN = *71*). **(H)** Performance summary of Model 3 at this threshold: sensitivity = *0.75*, specificity = *0.71*, PPV = *0.72*, NPV = *0.74*. **(I)** Feature-selection frequency across outer splits; bars show the percentage of splits in which each metric was retained by the final model. Genotype was selected in nearly all models, followed by *AUC*(*SB*), *s*(*H* − *SN*), *s*(*H* − *SB*), *C*_*l*_(*SN*), and *AUC*(*H*).

To further link the prelimbic microcircuit features with genotype and social behavior, we generated three machine learning models to compare their predictive performance (**Supplementary Figure S4, S5**). A summary of the predictive modeling frameworks, including inputs, targets, and evaluation indices, is provided in **Supplementary Table S2**. Below we reported model performance and key predictors for each model with all graph-theoretic measures considered (Figure 4).

#### Model 1 — Predicting genotype from calcium-activity and graph theory indices:

Using a cross-validated classifier, miniscope-derived network features predicted genotype (*Shank3*^*fx*^ vs WT) with an ROC–AUC = *0.76* (Figure 4A). At the default threshold, the normalized confusion matrix showed 61% of *Shank3*^*fx*^ and 85% of WT mice were correctly classified (accuracy = 0.73, sensitivity = *0.61*, specificity = *0.85*; Figure 4B). Precision for *Shank3*^*fx*^ was relatively high (PPV = *0.80*), whereas the negative predictive value was lower (*0.69*), indicating misclassifications were dominated by *Shank3*^*fx*^ mice incorrectly classified as WT. Feature-selection frequencies showed consistent contributions from *AUC*(*SB*), *s*(*H* − *SB*), *L*_*g*_(*SB*), *s(H–SN)*, and *C*_*l*_(*SB*) (>90% of folds), whereas *C*_*g*_(*SN*) appeared in fewer than one-third of folds (Figure 4C). Together, these results indicate that sociability stage features and spectral distances are the most informative for genotype prediction.

#### Model 2 — Predicting social-behavior differences from calcium-activity and graph theory indices:

Differences in social motivation were predicted from miniscope-derived network features. Across all outer test folds, the classifier achieved an ROC–AUC = *0.75* (Figure 4D). Relative to Model 1, Model 2 showed a more balanced discrimination between classes, suggesting less bias than Model 1. Model 2 had an accuracy of 71.5%, where 76% of mice exhibiting high social motivation were correctly classified and 67% of mice exhibiting low social motivation were correctly classified (sensitivity = 0.76, specificity = 0.67, PPV = 0.70, NPV = 0.74; Figure 4E).

Although Models 1 and 2 used the same set of graph-theoretic features, their variable-selection patterns differed. Model 2, which predicted social behavior, showed a more focused reliance on features capturing stage-dependent activity and network reconfiguration. Feature-selection frequencies highlighted spectral distance measures and calcium-activity (each selected in ≥ 99% of folds), indicating that stage-to-stage network reconfiguration and overall SB-stage activity were the most informative predictors (Figure 4F). In contrast, global clustering features, were selected in fewer than *40%* of folds, and global integration appeared less consistently than in the genotype model (38.4% vs 95.5%; Figure 4C, 4F).

Overall, only four of eleven graph-theoretic indices exceeded a *75%* selection frequency in the behavior model (compared with eight in Model 1), suggesting that atypical social behavior is supported by a narrower subset of dynamic network signatures. Notably, *AUC*(*SB*), *s*(*H* − *SB*), and *s*(*H* − *SN*) were shared high-value predictors in both models, indicating that while no graph-theoretic features were unique to behavioral expression, behavior prediction preferentially emphasizes dynamic, context-dependent network properties over stable topological features such as local clustering (*C*_*l*_) and global integration (*L*_*g*_).

#### Model 3 — Predicting social-behavior using genotype plus calcium-activity and graph theory indices:

Combining genotype with network features to predict social behavior yielded similar performance to Model 2, with an ROC–AUC = *0.76* (Figure 4G). Similar to Model 2, the confusion matrix was more balanced than Model 1 with an accuracy = 0.73, a PPV = 0.72, and NPV = 0.74 (sensitivity = 0.75, specificity = *0.71*; Figure 4H). Feature-selection frequencies (Figure 4I) showed genotype was retained in virtually all models (*99.9%*), followed by *AUC*(*SB*) (*84.3%*) and the spectral distances *s*(*H* − *SN*) and *s*(*H* − *SB*) (66.5% and *66.4%*). Additional contributors included *C*_*l*_(*SN*) (*50.1%*) and *AUC*(*H*) (*45.0%*), whereas *L*_*g*_(*SB*) (*27.2%*) and global-clustering indices *C*_*g*_ in SB/H/SN (*16–22%*) were less consistently selected. These patterns suggest that SB stage activity and state-dependent network reconfiguration (captured by spectral distances) provide complementary information for behavior prediction beyond genotype.

### In silico ablation analyses verified the importance of calcium-activity (*AUC*) and spectral distance (*s*) features for predicting genotype and behavior outcomes

#### Model 1—Predicting genotype from graph theory indices with removal of calcium activity and spectral distance (-AUC, -s):

**Figure 5:**
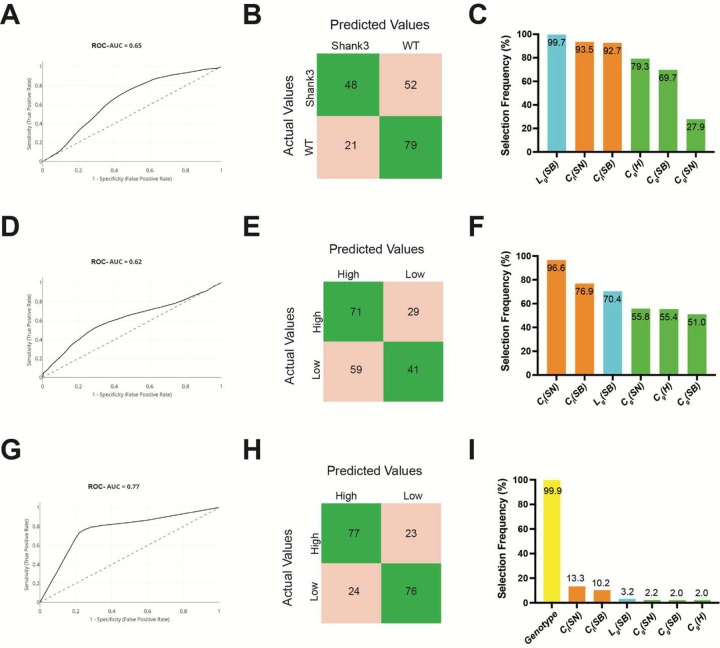
Combined in silico ablation of spectral distance and calcium activity features across machine learning models. Performance and feature-selection results are shown for ablation analyses in which all spectral distance features (*s*(*H* − *SB*), *s*(*H* − *SN*), *s*(*SB* − *SN*)) and all calcium-activity (*AUC*) features were removed, while other graph-theoretic metrics were retained. **(A–C) Model 1** (graph-theoretic indices → genotype). Panel A shows the ROC curve (ROC-AUC = 0.65), panel B shows the confusion matrix for WT versus *Shank3*^*fx*^ classification, and panel C shows feature-selection frequencies of the remaining predictors following combined feature removal. **(D–F) Model 2** (graph-theoretic indices → social behavior). Panel D shows the ROC curve (ROC-AUC = 0.62), panel E shows the confusion matrix for behavioral classification, and panel F shows feature-selection frequencies after removal of both spectral distance and calcium-activity features. **(G–I) Model 3** (graph-theoretic indices + genotype → social behavior). Panel G shows the ROC curve (ROC-AUC = 0.77), panel H shows the confusion matrix, and panel I shows feature-selection frequencies, highlighting the dominant contribution of genotype when network-derived spectral distance and calcium activity features are excluded.

While feature-selection frequencies indicate which predictors are preferentially used by the model, in silico ablation analyses directly test the dependence of model performance on specific feature sets by quantifying the effect of their removal. We first removed all spectral distance (*s*) and calcium-activity (*AUC*) features simultaneously from each model (Figure 5). Figure 5 (**panels A-C**) showed a clear reduction in the ability to distinguish WT and *Shank3*^*fx*^ mice after removal of calcium-activity and spectral distance features for Model 1. Model performance dropped substantially following in silico ablation, with an ROC-AUC = 0.65 compared to an ROC-AUC = 0.76 for the original model (Figure 5A vs 4A). Accuracy dropped by ~10 percentage points, with sensitivity dropping from 0.61 to 0.48 and specificity from 0.85 to 0.79 (Figure 5B vs 4B). In silico ablation was accompanied by a decrease in both PPV (70%) and NPV (60%) relative to the original model (PPV = 80%, NPV = 69%), driven by a 33% increase in misclassification of *Shank3*^*fx*^ mice as WT. Consistent with this loss of discrimination, feature-selection frequencies became increasingly concentrated on a limited subset of the remaining predictors rather than being broadly distributed across features, indicating that calcium-activity (*AUC*) and spectral distance (*s*) measures are not only frequently selected but also functionally necessary for maintaining robust genotype separation in Model 1, particularly for differentiating *Shank3*^*fx*^ from WT.

#### Model 2—Predicting social-behavior differences from graph theory indices with removal of calcium activity and spectral distance (-AUC, -s):

For Model 2 (social-behavior prediction), comparison of **panels D–F** between Figures 4 and 5 underscores the critical contribution of calcium-activity (*AUC*) and spectral distance (*s*) features to predicting social behavior. In the original model (Figure 4D–F), behavioral classification performance was relatively high ROC-AUC = 0.75, and feature-selection frequencies highlighted and spectral distance measures as the most consistently selected predictors. Following removal of these features, classification performance declined markedly (ROC-AUC = 0.62; Figure 5D). While both models predict high social motivation with similar sensitivity (0.71 for ablation model vs 0.76 for original model), the ablation model shows a substantially reduced PPV (0.55 vs 0.70) and NPV (0.59 vs 0.74), driven by a 79% increase in false positives, or instances where low social motivation was classified as high social motivation (Figure 5E). Correspondingly, feature-selection patterns became more concentrated on a narrower subset of remaining predictors and shifted toward less consistently selected graph-theoretic metrics, demonstrating that calcium activity levels and spectral distance features are key drivers of behavioral prediction and that their removal substantially weakens the model’s ability to capture abnormal social behavior, particularly in distinguishing low versus high social motivation (Figure 5F).

#### Model 3 — Predicting social-behavior using genotype plus graph theory indices with removal of calcium activity and spectral distance (-AUC, -s):

For Model 3 (behavior prediction with genotype included), comparison of **panels G–I** between Figures 4 and 5 reveals a marked shift in the contribution of graph-theoretic measures depending on feature availability. In the original model, with calcium-activity (*AUC*) and graph-theoretic indices (Figure 4), classification performance was high (ROC-AUC = 0.76), and feature-selection frequencies suggested network-derived features—particularly and spectral distance measures—provided predictive value beyond genotype. However, after removal of these features (Figure 5I), genotype became the only consistently selected predictor, with a slight increase in model performance (ROC-AUC = 0.77). Relative to the original Model 3, the confusion matrix showed fewer misclassifications (PPV = 0.76, NPV = 0.77), with reductions in both false positives (29 vs 24) and false negatives (25 vs 23) resulting in a 3.5-percentage-point increase in accuracy. This slight increase in performance suggests social behavior is largely explained by genetic differences and spectral distance measures provide limited additional information beyond genotype.

#### Predicting genotype and behavior outcomes with removal of only calcium activity features (-AUC):

To further isolate the contribution of calcium activity features, we performed partial in silico ablation analyses in which all calcium-activity (*AUC*) terms were removed while retaining spectral distance (*s*) measures and other graph-theoretic features (**Supplementary Figure 6**). Under this ablation, Model 1 retained moderate genotype classification performance (ROC-AUC = 0.72; **S6 panels A–C**), with feature-selection analysis indicating increased reliance on remaining integration and clustering measures. However, sensitivity dropped from 0.61 to 0.53 following ablation of calcium activity features, stemming from an increase in false negatives of ~20% (**Supplementary Figure S6B**). This suggests calcium activity features play a substantial role in distinguishing *Shank3*^*fx*^ from WT mice and without these features, more *Shank3*^*fx*^ mice are classified as WT. In contrast, Model 2 exhibited reduced performance in predicting social behavior following ablation of calcium activity features (ROC-AUC = 0.65; **S6 panels D–F**), with a 42% increase in false positives and a 20% drop in specificity (0.53). This suggests calcium-activity features help distinguish low from high social motivation, consistent with its role in Model 1 in differentiating *Shank3*^*fx*^ from WT mice. Notably, Model 3 remained robust to calcium-activity removal (ROC-AUC = 0.74; **S6 panels G–I**), with small differences in sensitivity (0.73), specificity (0.72), PPV (0.72), and NPV (0.73; **Figure S6H**). Genotype emerged as the most frequently selected predictor, followed by spectral distance features (48.5% and 47.3%; **S6I**), though at lower frequencies compared to the original model (66.5%, 66.4%; Figure 4I).

#### Predicting genotype and behavior outcomes with removal of only graph theory feature (-s):

To further isolate the contribution of spectral distance features, we performed partial in silico ablation analyses in which all spectral distance (*s*) terms were removed while retaining calcium-activity (*AUC*) and other graph-theoretic features (**Supplementary Figure S7**). Following this removal, Model 1 continued to show moderate genotype classification performance (ROC-AUC = 0.73), with similar a sensitivity (0.60), specificity (0.83), PPV (0.78), and NPV (0.67) as the original model (**S7 panels A–C**), suggesting performance remained robust to spectral distance removal. Model 2 again exhibited a reduced ability to predict social behavior (ROC-AUC = 0.67; **S7 panels D–F**), with only a slightly higher sensitivity (0.74) relative to calcium-activity ablation (0.70), though with the same specificity (0.53). Local clustering features had a higher selection frequency (97.7%, 83.0%) relative to the original model (89.4%, 71.9%) with Lg showing an ~86% increase in selection frequency (71.4%), suggesting these features cannot fully compensate for the loss of spectral distance measures. Similarly, Model 3 remained robust to spectral distance removal (ROC-AUC = 0.77; **S7 panels G–I**), with genotype emerging as the dominant predictor under this ablation and *AUC*(*SB*) selected less frequently (68.2%) than compared to the original model (84.3%).

## Discussion

In this study, we establish a multiscale link between prefrontal microcircuit functional connectivity, genotype, and social behavior by integrating single-cell resolution *in vivo* calcium imaging with network-theoretic and spectral analyses. Consistent with previous reports with *Shank3*^*fx*^ mice^12^, we observed reduced sociability and preference for social novelty accompanied by diminished neural somatic calcium activity in the prelimbic cortex. Beyond differences in neural activity levels, our network analyses revealed a distinct topological signature in *Shank3*^*fx*^ microcircuits characterized by increased shortest path length (*L*_*g*_) and reduced local clustering (*C*_*l*_), indicating impaired local coordination and reduced efficiency of information transfer. Interhemispheric abnormalities including under- and over-connectivity have been reported in human ASD functional imaging studies^32^, suggesting that microcircuit-level disruptions may scale up to the large-scale network differences observed clinically. Importantly, we further demonstrate that WT prelimbic microcircuits exhibit substantially greater stage-dependent reconfiguration across social contexts, whereas *Shank3*^*fx*^ prelimbic networks show blunted spectral separation between behavioral stages. This reduced network flexibility provides a mechanistic framework for understanding behavioral rigidity and diminished social adaptability commonly associated with ASD phenotypes.

Predictive modeling further demonstrated that these neural calcium activity and graph theory features carry behaviorally and genetically relevant information. Genotype and social behavior were both predictable from the same miniscope-derived network features, indicating a shared neural network signature linking genetic perturbation to behavioral phenotype. Feature importance converged across models, with sociability-stage calcium activity and cross-stage spectral distance measures consistently emerging as dominant predictors. This convergence suggests that reduced network integration and attenuated state-dependent reorganization jointly capture core aspects of Shank3-related circuit dysfunction.

In silico ablation analyses clarified the distinct roles of activity magnitude and dynamic network structure in supporting model performance. For genotype classification, removal of calcium activity features produced the largest performance decline, indicating that baseline differences in neural engagement are primary contributors to genotype discrimination. In contrast, prediction of social behavior relied on the joint contribution of calcium activity levels and spectral distance measures. Removing either feature class substantially degraded behavioral classification performance, demonstrating that both neural activation during social interaction and dynamic reconfiguration of network topology across behavioral stages are essential for capturing different social behavior. Together, these findings indicate that while genotype constrains baseline circuit architecture, behavioral variation emerges through context-dependent network dynamics.

When genotype information was included directly in behavioral prediction (Model 3), overall classification performance remained stable, and feature reliance shifted away from activity and spectral distance measures. This pattern suggests that genotype explains a substantial portion of behavioral variance, whereas network-derived features provide complementary information primarily when genotype is unknown. Notably, graph-theoretic metrics effectively served as proxies for genotype in models that excluded genetic labels, reflecting downstream circuit-level consequences of *Shank3* mutation. However, only a narrower subset of dynamic network signatures was consistently required for behavioral prediction, highlighting that not all genotype-associated circuit alterations translate directly into observable social behavior differences.

Collectively, these results support a hierarchical framework in which Shank3-related genetic perturbations shape baseline microcircuit topology, while behavioral variability is expressed through the capacity for dynamic reorganization of functional networks. Integrating graph-theoretic descriptors with spectral measures of cross-state network change therefore provides a powerful approach for dissecting how molecular perturbations propagate across scales to influence circuit function and behavior.

This work extends prior studies in several important ways. First, it demonstrates that microcircuit topology derived from single-cell resolution calcium imaging in freely behaving animals contains predictive information relevant to both genotype and social behavior, even with moderate sample sizes and relatively simple graph metrics. Second, it highlights the importance of behavioral context: features derived from the sociability stage and cross-stage spectral distances were repeatedly prioritized by machine learning models, underscoring the role of dynamic prefrontal engagement in social processing. Third, our analytical pipeline balances interpretability and generalization by incorporating conservative data-leakage controls, extensive cross-validation, and feature-selection frequency summaries rather than reliance on a single fitted model. The use of spectral distance metrics complements traditional graph indices by capturing global reorganization of network structure across behavioral states, an aspect not accessible through within-state topology alone.

Several limitations should be considered when interpreting these findings. The cohort size was moderate (WT = 9; *Shank3*^*fx*^ = 11), and external validation using independent datasets will be important for confirming generalizability. Genotype was used to define behavioral groupings due to sample-size constraints and inter-individual variability in social performance; larger datasets will be needed to distinguish low versus high social motivation independent of genetic labels. Additionally, Pearson correlation-based functional connectivity and binarization provide a tractable representation of network structure but cannot fully disentangle direct synaptic interactions from shared input or global state effects. Although threshold sensitivity analyses indicated that group-level conclusions were robust, future work incorporating partial correlations, state-space models, or GLM-based coupling approaches may further refine network inference. Finally, the present analyses are correlational and do not establish causal relationships between specific network features and behavior. Extending this framework to additional brain regions and ASD animal models, as well as incorporating experimental perturbations, will be critical for testing mechanistic hypotheses.

In summary, prelimbic microcircuit topology and its task-dependent reorganization provide a mechanistic substrate linking *Shank3*^*fx*^ genotype to social behavioral deficits. The consistent prominence of sociability-stage neural activity and cross-stage spectral distance measures highlights reduced flexibility of prefrontal networks in response to social cues as a defining feature of circuit dysfunction in *Shank3*^*fx*^ mice. These results identify dynamic microcircuit reconfiguration as a tractable target for future mechanistic and interventional studies aimed at restoring functional network adaptability in ASD-related disorders.

## Methods

### Animals

All experimental procedures were approved by the Institutional Animal Care and Use Committee of the National Institute on Drug Abuse (NIDA) and the University of Wyoming, and were conducted in accordance with the guidelines of the National Institutes of Health. *Shank3*^*fx*^ mice were originally obtained from the Jackson Laboratory (028800) *via* cryo recovery. Heterozygous *Shank3*^*fx*^ mice was bred to obtain homozygotes and maintained at the NIDA and the University of Wyoming. *Shank3*^*fx*^ mice have a *cre*-dependent FLEx switch allele containing an inverted *Shank3* PDZ domain (exons 13–16) flanked by inward-facing tandem *lox* sites (*lox2272*:*loxP*). This allele functions as a knockout allele in the absence of Cre recombinase, and homozygotes *Shank3*^*fx*^ exhibit autistic-like characteristics^12^. Mice were housed up to five mice per cage under a 12 h light/dark cycle and given *ad libitum* access to food and water.

### Stereotaxic injection

Young adult male homozygous *Shank3*^*fx*^ mice and C57Bl/6J (WT) mice (aged 3–6 months, weighing 25−35 grams) were used for experiments. Stereotaxic viral injection was performed unilaterally at the prelimbic region based on the published lab protocols^33, 34^. Mice were briefly anesthetized with 5% isoflurane in oxygen. The head was shaved and held on a stereotaxic stage with a heating pad at 35°C. Anesthesia was maintained with 1.5% isoflurane in oxygen throughout the surgery. An incision was made along the sagittal midline to expose the bregma and lambda. A 0.6 mm dental burr was used to drill a small hole through the skull at the coordinates A/P: +1.94 mm and M/L: 0.5 mm on the right hemisphere. AAV1-CaMKII-GCaMP6f (2.8 × 10^13^ GC/mL) virus was diluted with saline in a ratio of 1:1. Through a microliter syringe controlled by a micro-pump, 500 nL of virus was infused into the designated brain region (A/P: +1.94 mm, M/L: 0.5 mm, D/V: 1.75 mm) at a rate of 50 nL/minute. The virus was allowed to diffuse for additional 5−10 minutes before the skull skin was stitched for closure. Mice were allowed to recover for 14 days before proceeding to the next step.

### Gradient-index (GRIN) lens implantation

GRIN lens implantation into the mouse dorsal prelimbic cortex was performed according to the previously described lab protocols^33, 34^. Mice were anesthetized with an intraperitoneal injection of Ketamine/Xylazine (Ketamine: 100 mg/kg; Xylazine: 15 mg/kg) initially and maintained with additional doses of Ketamine (50 mg/kg) during the surgery. Craniotomy was performed at the coordinates A/P 1.94 mm and M/L 0.8 mm on the right side using a 1.2 mm drill burr. The brain tissue was aspirated layer-by-layer to the depth of 1.8 mm (measured from the bregma), with a blunt-end 27-G needle that was connected to an in-house vacuum system. The needle holder was connected to a robotic arm inclined to a 10° angle laterally, and was controlled by a custom-built software (https://github.com/liang-bo/AutoStereota)^35^. During the entire aspiration process, the exposed brain tissue was continuously rinsed with the artificial cerebrospinal fluid (ACSF) containing 124 mM of NaCl, 2.5 mM of KCl, 1.25 mM of NaH_2_PO_4_, 1.2 mM of MgCl_2_, 25 mM of glucose, 26 mM of NaHCO_3_ and 2.4 mM of CaCl_2_. ACSF was bubbled with a gas mixture of 95% O_2_ and 5% CO_2_ and filtered through a 0.2 μm filter. After the aspirated area was blood-free, a sterile GRIN lens (GRINTECH) was implanted into the well and secured with two layers of dental cement (first layer-Metabond, second layer-Duralay). The exposed GRIN lens was covered by a customized protection cap.

### Miniscope base mounting and in vivo calcium imaging during social behavior tests

All miniscopes used for the experiment were custom-built by Dr. Da-Ting Lin’s group at NIDA IRP^27, 28, 29^. After 3 weeks of GRIN lens implantation, a miniscope base was permanently mounted on the mouse skull. For base mounting, a miniscope fitted with a base was brought closer to the GRIN lens with the help of a custom-built motorized controller to obtain the best focal plane and the base was then affixed to the skull using dental cement. The main body of the miniscope was detached from the base after the dental cement had hardened. On the experimental day, mice were briefly anesthetized with 5% isoflurane in oxygen and the miniscope was fastened onto the base. Mice were allowed to recover from isoflurane for at least 30 minutes in their home cages before performing the *in vivo* calcium recordings.

A social behavior test was performed based on the previously published protocol^28^. The experiment was performed in an open square arena (42 cm × 42 cm × 30 cm) to facilitate calcium imaging where two small containers were placed near the two opposite corners (10 cm away from the sidewall of arena). The test was composed of three stages, habituation (H), sociability (SB), and social novelty (SN), each 10-minute long. During the H stage, the mouse was allowed to explore the two empty containers freely. In the SB stage, an age and gender matched (male) stranger mouse (stranger 1) was placed inside one of the containers whereas the other container remained empty. The SB stage measures the tendency of a subject mouse to spend more time exploring the container holding a social target compared to the empty container. During the SN stage, a second age and gender matched (male) stranger mouse (stranger 2) was placed inside the previously empty container. The SN stage measures the tendency of a subject mouse to spend more time exploring a novel social target compared to the relatively familiar social target.

For *in vivo* calcium imaging recording, the mouse was brought closer to the behavior arena and the miniscope was connected to a cable linked with the data acquisition computer system. Simultaneous recordings of the calcium imaging and mouse behavior were collected in two different computers with the aid of a custom-built calcium image recording software (NeuView) and a behavior recording software (FlyCap2, FLIR). The behavior recording was triggered by NeuView so that both calcium and behavioral recordings were temporally synchronized frame by frame. The recordings were performed at a rate of 10 frames per second. Each 10-minute stage was broken down into two 5-minute recording sessions to prevent excessive heating of the miniscope. Repetitive *in vivo* calcium imaging during social behavior test was performed every week for five times (SB1 to SB5, Figure 1A).

For behavior analysis, the mouse behavior was manually annotated frame by frame using a MATLAB annotation program (https://github.com/pdollar/toolbox) and the time spent by the subject mouse exploring the containers was calculated. It was considered that the subject mouse was exploring when it sniffed, bit, poked, or physically contacted the container.

### Calcium imaging data analysis

Calcium image processing: *In vivo* calcium imaging recorded from nine *Shank3*^*fx*^ and eleven WT mice were processed for data analysis. Calcium images were first registered using the motion correction toolbox NoRMCorre^36^. Then, a constrained non-negative matrix factorization (CNMF) based calcium image processing toolbox, CaImAn-MATLAB^37, 38, 39^, was used to extract fluorescent calcium signals. We manually added additional seeds as necessary based on the correlation image calculated by the correlation of neighboring pixels of calcium images. Calcium traces and referred events were calculated using the functions of CaImAn toolbox. For each neuron, the amplitude of calcium traces was normalized to the maximum of values, and then the normalized values were summed up and divided by the number of seconds of imaging to obtain the average Ca^2+^ signal per *100* seconds (referred to as “area under curve per *100* seconds”, or s). To calculate frequency, the calcium events for each neuron were calculated by a widely used deconvolution method^40^ and binarized with a threshold of 5 standard deviations. The averaged event numbers per second were then calculated. For each neuron, the averaged s during each stage (i.e., H, SB, and SN stages) were also calculated.

#### Cell maps, network construction, and hub neurons

Based on the neurons and calcium activity traces, functional microcircuits were constructed for each imaging session including behavioral stages (H, SB, and SN). Each identified neuron was treated as a node in the network, and its spatial location was determined from the centroid coordinates of the corresponding calcium region of interest.

To illustrate the spatial distribution of neurons, cell maps were generated by plotting the spatial coordinates of all neurons within the imaging field, providing a two-dimensional representation of the anatomical layout of the microcircuit. In these maps (**Supplementary Figure S1**), each node corresponds to a single neuron, preserving the relative spatial organization of neurons within the field of view.

To quantify functional interactions among neurons, pairwise correlations were computed using the normalized calcium activity traces (*ΔF/F*) across the recording period within each behavioral stage. For each neuron pair (*i, j*), the correlation coefficient measured the degree of coordinated activity and served as an estimate of functional coupling. The resulting correlation matrix summarized pairwise functional relationships among all neurons within a microcircuit.

To construct a network representation suitable for graph-theoretic analysis, the correlation matrix was converted into a binary adjacency matrix *A= [a*_*ij*_*]*. Correlation values exceeding a predefined threshold were classified as functional connections and assigned *a*_*ij*_ = 1, while correlations below the threshold were assigned *a*_*ij*_ = 0. This binarization resulted in an undirected, unweighted graph in which nodes represent neurons and edges represent significant functional interactions. The resulting adjacency matrix provided a consistent representation of functional connectivity across microcircuits, behavioral stages, and experimental groups.

Within each constructed network, node degree was quantified as the number of connections per neuron. To identify highly connected nodes, we employed an empirical tail criterion based on the observed degree distribution within each microcircuit. Specifically, for neuron *i* with degree *d*_*i*_, we defined a rank-based p-value

$$p_{i}=\frac{1}{N}\sum_{j=1}^{N}I\left(d_{j}\geq d_{i}\right)$$

where *N* is the total number of neurons in the network and *I*(·) denotes the indicator function. This quantity represents the proportion of neurons whose degree is at least as large as that of neuron *i*, providing a rank-based measure of relative hubness without assuming a generative null model. Neurons with *p*_*i*_ ≤ 0.05 were classified as hub neurons, corresponding approximately to the upper 5% tail of the degree distribution within each network. This empirical definition yields a consistent within-network ranking of highly connected nodes and was used for visualization purpose only.

### Graph theory analysis to quantify functional connectivity of the prelimbic microcircuit

Based on the networks constructed above, graph-theoretic indices were computed to quantify microcircuit topology and compare network organization across experimental conditions. Definitions and mathematical formulations of all graph-theoretic indices are provided in **Supplementary Table S1**.

We computed indices that characterized its organization—global integration (*L*_*g*_), local segregation (*C*_*l*_), and network-wide clustering (*C*_*g*_)—and we compared networks with the same labeled nodes under different conditions using a spectral difference, *s*, derived from their Laplacian eigenvalues.

*L*_*g*_ was calculated by first computing the average shortest distance for all nodes to all other nodes (Figure. 3A, 3B), then taking the mean of the resulting values:

$$L_{g}=\frac{1}{N\left(N-1\right)}\sum_{i,j\in Vi\neq j}\text{dist}\left(i,j\right),$$

Where *dist* is the shortest path between node *i* and *j*, and *N* is the number of nodes. *L*_*g*_ measures the integration of a neuronal graph and how efficiently information can flow within a graph.

For a given node *v*, its local clustering coefficient is determined by:

$$C_{v}=\frac{\text{Number}\;\text{of}\;\text{closed}\;\text{triplets}}{\text{Number}\;\text{of}\;\text{all}\;\text{triplets}}$$

Where triplets refer to all possible combinations of three neighbors of *v*, both opened and closed^41^. An example of closed triplets in a graph is illustrated in Figure 3D. *C*_*l*_ is the average of all local clustering coefficients, defined by:

$$C_{l}=\frac{1}{N}\sum_{v\in V}C_{v}$$

*C*_*g*_ is global clustering coefficients, provides the overall level of connectivity within a graph^41^. *C*_*g*_ is defined by:

$$C_{g}=\frac{\text{Number}\;\text{of}\;\text{closed}\;\text{triplets}\;\text{in}\;a\;\text{graph}}{\text{Number}\;\text{of}\;\text{all}\;\text{triplets}\;\text{in}\;a\;\text{graph}}$$

The Laplacian matrix L for a given graph is defined as

L=D−A

where *D* = diag*(d*_*1*_*, d*_*2*_*, …, d*_*N*_*)* is a diagonal matrix and *d*_*i*_ is the degree of the *i*th vertex with *N* total nodes in the graph, and *A* is the adjacency matrix of the graph. Since the spectrum of the representation matrix of a network, the Laplacian matrix, carries information about its structure, comparing spectral matrices provides metrics for comparing structural changes in networks. Because the Laplacian matrix *L* is real and symmetric, all the eigenvalues are real and non-negative. The set consisting of the eigenvalues of *L* is called the spectrum of *L*. Assuming two graphs with the same nodes have the corresponding Laplacian matrices *L*_*1*_ and *L*_*2*_, which have the corresponding eigenvalues *λ*_*1*_*, λ*_*2*_*, …, λ*_*N*_ and *ω*_*1*_*, ω*_*2*_*, …, ω*_*N*_, respectively, we take the index *s*, the spectral distance which is the Euclidean distance between the two sets of eigenvalues normalized by the square root of the number of neurons in the graphs^42^, as a measure to compare these two graphs:

$$s=\sum_{i=1}^{N}\frac{1}{N}\sqrt{{\left(\lambda_{i}-\omega_{i}\right)}^{2}}$$

In comparing networks, one of the key challenges lies in quantifying structural similarity when nodes of two networks are the same, but their connectivity patterns differ. Here, we used the spectral distance (*s)*, computed from the differences between Laplacian eigenvalues, to quantify network similarity. This measure reflects global organization rather than individual link differences (Figure 3G).

### Machine learning analysis to predict mouse genotype and behavioral outcomes

We trained three supervised models using miniscope-derived network features to identify graph theoretic measures predictive of genotypic and social differences (**Supplementary Figures S4 — S5**). Predictors included session-level graph indices — characteristic path length *L*_*g*_, local clustering *C*_*l*_, global clustering *C*_*g*_— for H, SB, and SN, session activity *AUC(H/SB/SN)* from normalized *ΔF/F*, and spectral distances *s*(*H* − *SB*), *s*(*H* − *SN*), *s*(*SB* − *SN*).

#### Genotype prediction (Machine learning Model 1):

In the first model, graph theoretic indices were used to predict genotype, enabling identification of neural features that directly distinguish genetic groups (*Shank3*^*fx*^ vs WT).

#### Behavior prediction (Machine learning Model 2):

In the second model, graph-theoretic indices were used to predict genotype-associated behavioral differences, defined as high vs low social motivation derived from sociability/novelty scores. The composite social motivation score, *S*_avg_, was computed per session as the mean of sociability (SB) and social novelty (SN). To ensure we captured differences in social motivation linked to autism, an ROC analysis was conducted to identify the *S*_avg_ threshold that best discriminates *Shank3*^*fx*^ from WT mice. The value nearest the top-left corner of the ROC curve was used to binarize *S*_avg_ into high vs low social motivation. This thresholding step used only SB/SN and genotype (no imaging features), was set once, and was not tuned within cross-validation (Supplementary Methods).

#### Behavior prediction (Machine learning Model 3):

Similar to Model 2, Model 3 used graph-theoretic indices to predict genotype-associated behavioral differences but with genotype included as an additional predictor (i.e., graph-theoretic indices + genotype). Since the behavioral differences are strongly associated with genotype, this model helps determine if genotype alone is predictive of behavior, or if graph indices add information beyond genotype.

#### Preprocessing and feature reduction:

Graph indices were transformed to stabilize variance. Right-skewed variables (e.g., *s*(·), *C*_*l*_) were log-transformed, while strongly skewed *C*_*g*_ features used inverse transformation. To reduce redundancy, highly correlated predictors were pruned using with a cutoff = *0.8*. Univariate outliers were winsorized to fall within 3 median absolute deviations, while multivariate outliers were screened by Mahalanobis distance with the cutoff of $\chi_{p;0.975}^{2}$. Pre-processing was done once across the full dataset to provide a consistent feature space and facilitate interpretation. The final candidate subset used for machine learning was: *AUC*(*H*), *AUC*(*SB*), *AUC*(*SN*), *s*(*H* − *SB*), *s*(*H* − *SN*), *L*_*g*_(*SB*), *C*_*l*_(*SB*), *C*_*l*_(*SN*), *C*_*g*_(*H*), *C*_*g*_(*SB*), *C*_*g*_(*SN*).

#### Models and tuning:

We used a random forest classifier with mtry tuned by cross-validation over a grid up to ⌈√p⌉, where p is the number of features (R package caret). Recursive feature elimination^43^ was used for feature selection to identify the optimal subset of features for learning. Feature-selection frequencies were aggregated across cross-validation folds to summarize variable stability.

#### Cross-validation and enumeration:

Nested cross-validation was used to provide an unbiased estimate of model performance and prevent overfitting (**Supplementary Figure S4**). To respect mouse-level grouping and maintain genotype balance, we used a leave-two-pairs-out outer loop holding out two WT and two *Shank3*^*fx*^ mice per fold (all sessions from each held-out mouse were assigned to the test set). Within each outer training set, an inner CV performed hyperparameter tuning and RFE using a leave-one-pair-out scheme. This approach prevented data leakage such that the outer test set remained unseen until the final evaluation for that fold.

#### Evaluation:

Model performance across varying decision thresholds was visualized using an average ROC curve, with performance summarized by the area under the curve (ROC-AUC). Normalized confusion matrices were used to summarize classification performance with the default threshold. Secondary metrics derived from confusion matrices included accuracy, sensitivity, specificity, positive predictive value (PPV), and negative predictive value (NPV). Further details can be found in Supplementary Information for Methods and **Supplementary Table S2**.

#### In silico ablation analyses:

To assess the contribution of specific feature groups to model performance, we conducted a series of ablation analyses on the baseline machine learning models. Because spectral distance (*s*) and calcium activity (*AUC*) based features were among the most frequently selected predictors in the baseline feature-selection procedures, we focused on evaluating their impact on classification performance. For each model, we performed a full ablation in which all spectral distance and features were simultaneously removed from the feature set, while all other preprocessing steps, model architectures, hyperparameters, and training procedures were held fixed. Models were then retrained and evaluated using the same cross-validation framework and performance metrics as in the baseline analyses. This approach allowed us to quantify changes in predictive performance attributable to the removal of these feature groups and to examine how feature-selection patterns shifted under ablation.

#### Software and reproducibility:

Analyses used R (version 4.4.2) with packages caret^44^, randomForest^45^, pROC^46^, performance^47^, datawizard^48^, plotly^49^ and the tidyverse^50^ ecosystem. Custom Matlab (R2024a) scripts were used to construct graph indices. Full code and session information are provided in the Supplementary Information.

### Statistics

We used a Linear Mixed-Effects Model (LMM) to analyze repeated measures data, specifically the graph-theoretical descriptors (*AUC*, *C*_*g*_, *C*_*l*_, and *L*_*g*_). To account for the hierarchical structure of the data, we utilized a random intercept model where each subject was assigned an individual baseline level. The model included genotype and behavioral stage as fixed effects, along with their interaction. Post-hoc inferences were conducted using estimated marginal means to decompose significant effects. For derivative variables S1 (refers to the percentage time on exploring social target during the SB stage, Figure 1C), S2 (refers to the percentage time on exploring the novel social target during the SN stage, Figure 1C), *s*(*H* − *SB*), *s*(*H* − *SN*), *s*(*SB* − *SN*), that represent spectral differences (*s*) calculated across stages, the fixed effects were restricted to genotype only.

## Supplementary Material

**Supplementary Figure S1. Cell maps from all WT and *Shank3***^***fx***^ **mice** (related to Figure 1).

**(A)** Spatial cell maps (CaImAn footprints/centroids) from all WT mice (*n = 11*).

**(B)** Spatial cell maps from all *Shank3*^*fx*^ mice (*n = 9*).

Note: All cell maps summarize the detected neurons from one session/mouse; acquisition and preprocessing details were provided in Methods.

**Supplementary Figure S2. Social behavior analysis of WT mice and *Shank3***^***fx***^ **ASD mouse model** (related to Figure 1).

**(A)** Exploration time during social behavior tests SB1-SB5 for wild type (WT) mice. The social behavior test consists of habituation (H), sociability (SB), and social novelty (SN) stages, each lasting 10 minutes. E1 and E2 indicate empty containers; S1 and S2 indicates social targets.

**(B)** Exploration time during social behavior tests SB1-SB5 for *Shank3*^*fx*^ mice. Mann-Whitney test. *, *p* < *0.05*; **, p < 0.01; ****, *p* < *0.0001*; NS, not significant.

**Supplementary Figure S3. Cell maps and hub-neuron subnetworks across behavioral stages in WT and *Shank3***^***fx***^ **mice** (related to Figure 2).

**(A)** Representative examples from two WT mice (WT-05 and WT-10).

**(B)** Representative examples from two *Shank3*^*fx*^ mice (Shank3-02 and Shank3-05). For each mouse, neuronal spatial cell maps (left) and corresponding hub-neuron subnetworks (right) are shown for three behavioral stages: H, SB, and SN. In the cell maps, each dot represents a neuron, with hub neurons highlighted in red and non-hub neurons shown in blue. Hub-neuron subnetworks display only hub nodes and their functional connections, illustrating differences in hub connectivity patterns across stages and genotypes. WT microcircuits show pronounced stage-dependent reorganization of hub connectivity, particularly during social interaction, whereas *Shank3*^*fx*^ microcircuits exhibit altered hub distributions and reduced reconfiguration across behavioral stages.

**Supplementary Figure S4. Nested cross-validation scheme** (related to Figure 4). The outer loop exhaustively enumerates *1,980* test folds by selecting two mice per genotype as the outer test set (green), with the remaining animals used as outer training data (blue). For each outer fold, an inner leave-pair-out cross-validation is run on the outer-training set: in each inner fold, one genotype-balanced pair is held out for validation (yellow) and the rest used for inner training (dark blue). The inner loop selects features and optimal hyperparameters. Final performance is computed only on the outer test set and then averaged across all *1,980* folds. Mouse icons denote genotype (WT = black; *Shank3*^*fx*^ = gray). This design preserves class balance and prevents information leakage.

**Supplementary Figure S5. Correlation of network indices** (related to Figure 4). Pearson correlation matrix (color scale −1 to 1) for the candidate graph-theoretic features. Blocks reveal strong within-family dependencies: *L*_*g*_ across networks are highly correlated with one another (e.g., *r* ≈ 0.9); clustering measure *C*_*l*_ is positively correlated (*r* ≈ 0.8); and spectral distances sharing a common network, particularly *s*(*H* − *SN*), with *s*(*SB* − *SN*), are also highly correlated (*r* ≈ 0.9). As for between-family dependencies, *L*_*g*_ is strongly negatively correlated with clustering *C*_*l*_ within each social behavior test stage (*r* ≈ −0.8). These dependencies motivated pruning of redundant variables in the data-preparation step.

**Supplementary Figure S6. In silico ablation analysis of calcium-activity (*AUC*) feature across machine learning models** (related to Figure 5).

Performance and feature-selection results are shown for partial ablation analyses in which all calcium-activity (*AUC*) features were removed while spectral distance (*s*) and other graph-theoretic features were retained.

(A–C) Model 1 (graph-theoretic indices → genotype). Panel A shows the ROC curve (ROC-AUC = 0.72), panel B shows the confusion matrix for WT versus *Shank3*^*fx*^ classification, and panel C shows feature-selection frequencies of the remaining predictors following removal.

(D–F) Model 2 (graph-theoretic indices → social behavior). Panel D shows the ROC curve (ROC-AUC = 0.65), panel E shows the confusion matrix for behavioral classification, and panel F shows feature-selection frequencies after removal of features.

(G–I) Model 3 (graph-theoretic indices + genotype → social behavior). Panel G shows the ROC curve (ROC-AUC = 0.74), panel H shows the confusion matrix, and panel I shows feature-selection frequencies, highlighting the increased contribution of genotype following removal of calcium-activity features.

**Supplementary Figure S7. In silico ablation analysis of spectral distance (*s*) feature across machine learning models** (related to Figure 5).

Performance and feature-selection results are shown for partial ablation analyses in which all spectral distance features (*s*) were removed while calcium-activity (*AUC*) and other graph-theoretic features were retained.

(A–C) Model 1 (graph-theoretic indices → genotype). Panel A shows the ROC curve (ROC-AUC = 0.73), panel B shows the confusion matrix for WT versus *Shank3*^*fx*^ classification, and panel C shows feature-selection frequencies of the remaining predictors following spectral distance removal.

(D–F) Model 2 (graph-theoretic indices → social behavior). Panel D shows the ROC curve (ROC-AUC = 0.67), panel E shows the confusion matrix for behavioral classification, and panel F shows feature-selection frequencies after removal of spectral distance features.

(G–I) Model 3 (graph-theoretic indices + genotype → social behavior). Panel G shows the ROC curve (ROC-AUC = 0.77), panel H shows the confusion matrix, and panel I shows feature-selection frequencies, highlighting the dominant contribution of genotype when spectral distance features are excluded.

Supplementary Tables:

**Supplementary Table S1.** Definitions and formulas of graph-theory indices used for network analysis. Detailed descriptions of calcium activity (*AUC*), shortest path length (*L*_*g*_), clustering coefficients (*C*_*l*_ and *C*_*g*_), and spectral distance (*s*) are provided to illustrate how each metric quantifies neural network activity and topology.

**Supplementary Table S2.** Summary of three predictive machine learning modeling frameworks linking graph theory indices, genotype, and behavior.

Supplementary Files

This is a list of supplementary files associated with this preprint. Click to download.

- SupplementaryInformationJan282026.docx

## Data Availability

Lead contact: Requests for further information and resources should be directed to and will be fulfilled by the lead contact, Yun Li (yli30@uwyo.edu).
